# Engineered PVA Hydrogel as a Universal Platform for Developing Stable and Sensitive Microbial BOD-Biosensors

**DOI:** 10.3390/bios16010042

**Published:** 2026-01-04

**Authors:** Anastasia Medvedeva, Aleksandra Titova, Anna Kharkova, Roman Perchikov, George Gurkin, Lydmila Asulyan, Leonid Perelomov, Maria Gertsen, Vyacheslav Arlyapov

**Affiliations:** 1 Research Center “BioChemTech”, Tula State University, Lenin Avenue, 92, Tula 300012, Russia; 2 Department of Chemistry, Tula State University, Lenin Avenue, 92, Tula 300012, Russia; 3 Laboratory of Biogeochemistry, Faculty of Natural Sciences, Tula State Lev Tolstoy Pedagogical University, Lenin Avenue, 125, Tula 300026, Russia; perelomov@rambler.ru (L.P.); mani.gertsen@gmail.com (M.G.)

**Keywords:** polyvinyl alcohol, hydrogels, biosensor, BOD, two-mediator system, *Blastobotrys adeninivorans*

## Abstract

Polyvinyl alcohol (PVA) hydrogels modified through radical polymerization under UV irradiation and Ce^4+^ ion treatment were investigated as a potential platform for developing highly sensitive biosensors for rapid biochemical oxygen demand analysis in water. These modifications enhance PVA physicochemical properties, including mechanical strength, stability, and biocompatibility, making it promising for immobilizing microorganisms in bioanalytical systems. A dual-mediator biosensor system using ferrocene (FC) and neutral red (NR) was developed with yeast *Blastobotrys adeninivorans* immobilized in modified PVA. The FC+NR–*B. adeninivorans*–PVA–Ce^4+^ system exhibited high sensitivity (linear range of 0.1–3.81 mgO_2_/dm^3^), selectivity, and operational stability (up to 37 days service life), outperforming existing analogs. Testing with wastewater confirmed strong correlation with standard BOD_5_, highlighting the potential for monitoring water quality. The described radical modification method is a simple and effective approach for creating sensitive and stable biosensors. It opens up new possibilities for environmental monitoring technology.

## 1. Introduction

Polyvinyl alcohol (PVA) hydrogels are versatile nonionic hydrophilic with unique properties [1]. PVA exhibits excellent water retention, maintains its structural integrity, recovers after mechanical stress, and is environmentally safe [2,3,4]. PVA is widely used in medicine [5], packaging materials [6], agriculture [7], as well as in various sensors for monitoring [8,9] and other fields. Frequently utilized as a support matrix for immobilizing enzymes and cells, PVA can have its catalytic performance enhanced. By functionalizing the polymer or combining it with other materials, key biocatalyst properties such as operational stability and scalability are improved [10,11,12]. This is exemplified by the commercial PVA-based product LentiKats^®^ (Prague, Czech Republic), known for its favorable mechanical characteristics in immobilization technology [13].

PVA cryogels have gained significant popularity as carriers for cell immobilization, largely because of their exceptional operational stability. Mechanism behind its gel formation via freeze–thaw cycles descried in work [14]. These freeze–thaw PVA hydrogels have been successfully applied, for instance, to immobilize a wide range of microorganisms, including various bacterial, yeast, and fungal cultures, have been encapsulated within this matrix [15,16,17,18]. Despite their advantages, the immobilization process using PVA cryogels presents challenges. Cells suspended in the PVA solution must endure multiple freeze–thaw cycles, and the material’s inherent viscosity and high degree of water swelling can complicate carrier preparation. To address these issues and enhance cell viability, compounds such as salts, sugars, or specific cryoprotectants are often incorporated to improve solubility and offer protection during matrix formation [19]. Furthermore, the inclusion of additives like sodium alginate, powdered activated carbon, silica, or zeolite powder has been shown to improve the mechanical strength and adhesive properties of PVA [19]. It is critical, however, that these supplements do not disrupt the low-temperature gelation process or negatively alter the physical characteristics of the final PVA matrix. Consequently, an optimal PVA hydrogel for immobilizing living cells must achieve a dual purpose: maintaining sufficient mechanical integrity while also ensuring high cell survival rates throughout the fabrication procedure.

Free-radical polymerization is widely used in the chemical modification of polyvinyl alcohol (PVA) for the synthesis of hydrogels, membranes, and coatings [20]. Cerium ammonium nitrate is a common redox initiator for this process, particularly effective for grafting onto polymers containing hydroxyl groups, such as chitosan or cellulose [21,22]. The Ce(IV)/Ce(III) redox cycle generates free radicals that initiate chain growth. Cerium ammonium nitrate offers several advantages, including green synthesis conditions (aqueous media), low-temperature operation, rapid initiation with short induction periods, and high versatility in grafting and crosslinking applications. Its effectiveness with hydroxyl-rich substrates like cellulose leads to functional polymers with enhanced stability, conductivity, and unique nanostructures, all while avoiding the use of harsh organic solvents. After polymerization, residual cerium ions remain as impurities and must be removed, typically by thorough washing of the polymer with water or ethanol. Our research group has previously developed several laboratory models of biosensors based on bacterial and yeast cells immobilized in PVA crosslinked with N-vinylpyrrolidone (N-VP), where hydrogel provide maintaining sufficient mechanical integrity and ensuring high cell survival rates throughout the fabrication procedure. For instance, we created a biochemical oxygen demand (BOD) biosensor using an association of yeast microorganisms *Pichia angusta*, *Arxula adeninivorans*, and *Debaryomyces hansenii* immobilized in N-VP -modified PVA [23]. The biosensor demonstrated a broad substrate detection spectrum and enabled water analysis across a wide BOD range (2.4–80 mg/dm^3^) with high correlation to the standard method (R = 0.9988). In study [24], we successfully immobilized bacteria isolated from activated sludge in a similar hydrogel. This biosensor allowed analysis of water samples initially classified as clean (BOD range 0.05–5.0 mg/dm^3^) with a correlation of R^2^ = 0.9990 to the standard method. Furthermore, in work [25], the same hydrogel was effectively used to immobilize *Paracoccus yeei* bacteria, creating a highly sensitive BOD biosensor with a lower detection limit of 0.05 mg/dm^3^. In this study, we propose using cerium-cross-linked PVA for microbial immobilization—a significantly simpler alternative to grafted PVA with N-VP, as it avoids the need for arduous purification of toxic unreacted N-VP monomer and poly N-VP homopolymer, a process for which dialysis is often inadequate. We demonstrate the utility of this straightforward *B. adeninivorans* immobilization matrix in the development of a mediator-based biosensor for biochemical oxygen demand (BOD) analysis.

The accuracy of BOD_5_ analysis using standard methods relies on calibration solutions, which are also essential for calibrating rapid assessment systems, including amperometric mediator biosensors. In such biosensors, immobilized microorganisms oxidize organic compounds from the sample, reducing an electron transport mediator. The reduced mediator is then oxidized on the electrode surface at a fixed potential, generating a current increase proportional to the concentration of oxidizable substrates. This establishes a direct quantitative relationship between the analytical signal and the BOD_5_ of the sample. Yeasts like *B. adeninivorans* are promising for their broad substrate range and environmental robustness. However, their thick cell wall limits electron transfer in single-mediator systems. This challenge can be addressed using dual-mediator systems, where one mediator interacts with intracellular cofactors and another shuttles electrons to the electrode [26]. For optimal sensitivity, the mediators must have complementary kinetics to avoid competition. This principle was successfully applied in a previous study using the “ferrocene–neutral red” pair with *B. adeninivorans* yeast, resulting in a highly sensitive biosensor suitable for low BOD concentrations found in surface waters [27].

**Figure 1:**
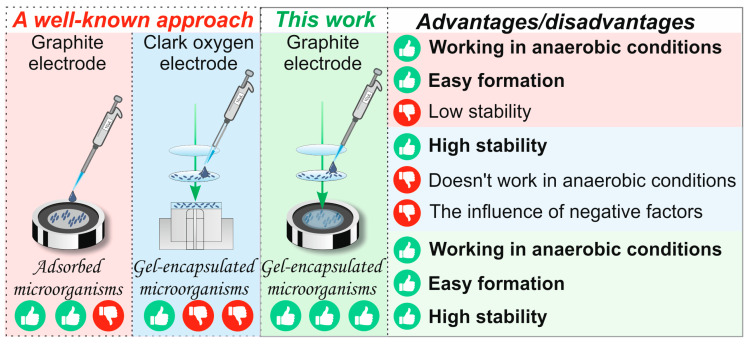
The biosensor fabrication approach employed in this study.

Mediator-based biosensors are particularly promising for environmental monitoring as their signal is independent of dissolved oxygen, allowing for use in anaerobic conditions. They offer rapid analysis (within minutes), high sensitivity, and potential for miniaturization. The proposed cerium-cross-linked PVA hydrogel provides an ideal, simplified platform for immobilizing such microbial biocatalysts, facilitating the creation of stable, reproducible, and sensitive BOD biosensors without the complexities associated with graft copolymer supports (Figure 1).

PVA hydrogels obtained by radical polymerization are an innovative material that opens up new horizons in the field of creating highly sensitive sensors for rapid analysis of water pollution. This polymerization method makes it possible to obtain hydrogels with specified properties and improved characteristics, which opens up new horizons for the development of environmentally friendly technologies. The absence of the need for additional crosslinking reagents makes these hydrogels more durable and resistant to various influences, as shown in Figure 1. Thus, PVA hydrogels obtained by radical polymerization are a unique material for creating highly sensitive sensors capable of quickly and accurately detecting water pollution.

## 2. Materials and Methods

### 2.1. Modification of Polyvinyl Alcohol by UV Irradiation (PVA-UV)

The irradiation was carried out using an ultraviolet lamp with a wavelength of λ = 254 nm, 50/60 Hz, model VL-6.LC (Vilber, Paris, France). For this purpose, 5 mL of 5–10% aqueous solution of polyvinyl alcohol (PVA, molecular weight M = 78,000, hydrolysis 98.0~98.8 mol/%, viscosity 4.0~5.0 mPa.s, FerakBerlin, Berlin, Germany) were placed in a Petri dish with a diameter of 9 cm. The irradiation was carried out for 15–60 min at a distance of 10 cm in an atmosphere of atmospheric oxygen.

### 2.2. Modification of Polyvinyl Alcohol Using Ce^4+^ Ions (PVA-Ce^4+^) as an Initiator of Crosslinking

To prepare a polymer using Ce^4+^ as a crosslinking initiator, 0.8 mL of an aqueous solution of cerium ammonium nitrate (NH_4_)_2_Ce(NO_3_)_6_ (T = 0.1 g/mL) was added to 20 mL of a 5% aqueous solution of PVA (M = 78,000, hydrolysis 98.0~98.8 mol/%, viscosity 4.0~5.0 mPa.s, FerakBerlin, Berlin, Germany) at a temperature of 55 °C and constant stirring in a nitrogen atmosphere for 3 h.

### 2.3. Determination of Viscosity, Proportion of Crosslinked Polymer, and Degree of Swelling in Aqueous Solutions

The viscosity was determined using capillary viscometry. Viscometer VTL-2 (Labtex, Moscow, Russia). was used for viscosity measurements. Relative viscosity was experimentally determined by comparing the expiration time of the polymer solution with that of the solvent, following a known procedure.

The proportion of crosslinked polymer in the film was determined by extracting it. To do so, a sample of the film was weighed, placed in water, and stirred for 4–6 h at a temperature of 50–60 °C. After that, the water was drained and the swollen film was dried in a drying cabinet at the same temperature until it reached a constant weight. This process was repeated several times until the mass of the sample no longer changed after drying.

The degree of swelling in aqueous solutions. To determine the degree of swelling, a sample of dry gel was weighed on an analytical balance and a vessel containing a swelling medium (distilled water, salt solution) was lowered for a certain time. After that, the swollen gel was filtered and weighed. The degree of swelling was determined by the Formula (1).

(1) $$Q=\frac{m-m_{0}(1-\gamma )}{m_{0}(1-\gamma )}\;\;,$$

where *Q*—is the degree of swelling of the hydrogel, g/g; *m*—is the mass of the swollen sample, g; *m*_0_—is the initial mass of the gel sample, g; *γ*—is the moisture content of the gel sample, mass fraction.

### 2.4. Scanning Electron Microscopy (SEM)

The surface of the PVA hydrogel samples was examined using a Hitachi TM 4000 Plus scanning electron microscope (Hitachi High-Tech Corporation, Tokyo, Japan). The studied samples were fixed on carbon tape and an aluminum table. Images were acquired in the secondary electron mode at an accelerating voltage of 15 keV without spraying. SEM investigations, coupled with an energy-dispersive X-ray spectroscopy (SEM-EDX), were performed using an EDX attachment (Bruker, Karlsruhe, Germany) at an accelerating voltage of 20 kV. The pore size was analyzed using the TM4000 microscope software version 1.2.0.3, and at least 10 micrographs were marked for each sample.

### 2.5. IR Spectroscopy

A Fourier spectrometer FMS 1201 manufactured by Monitoring LLC, Saint Petersburg, Russia, was used to register the infrared spectra of the starting materials and reaction products. Disks with potassium bromide (KBr) were prepared to obtain the spectra of solids. The method of preparing the discs was as follows: a sample of a solid substance (1–3 mg) was thoroughly mixed in a mortar with spectroscopically pure potassium bromide (150–200 mg). The mixture was then pressed under pressure 7.5–10 t/cm^2^ for 2–5 min. The spectrum of the obtained sample was taken relative to the air.

### 2.6. Raman Spectroscopy

The Raman spectra were obtained using an M532 Raman microscope manufactured by (Spektr-M, Chernogolovka, Russia) which was developed in Russia. Exciting radiation with a wavelength of 532 nm was generated by a He–Ne laser.

### 2.7. NMR-Spectroscopy

Nuclear magnetic resonance (NMR) spectra were obtained using (Bruker Corporation, Visp, Switzerland) Fourier 300HD spectrometers operating at frequencies of 300.1 MHz for protons ^1^H and 75 MHz for ^13^C. Chemical shifts of NMR of ^1^H and ^13^C were determined relative to the solvent signals. The Bruker Topspin 2.1 software package was used to process NMR spectra. All analyzed samples were dissolved in heavy water D_2_O (SOLVEX, Moscow, Russia).

### 2.8. Determination of Physico-Mechanical Properties of Polymer Films Based on Polyvinyl Alcohol

The determination of the physico-mechanical properties of polymers based on PVA was carried out on a universal bursting machine “INSTRON” (INSTRON, Norwood, MA, USA). Sample preparation and calculation of physical and mechanical characteristics were carried out according to the methods of the manufacturer. Each sample was measured 5 times.

### 2.9. Toxicity Assessment

Polymer toxicity was evaluated via a standardized bacterial bioluminescence inhibition test. Aqueous extracts of PVA-based polymers were prepared for analysis by immersing 1 g of the polymer in 5 mL of distilled water. Toxicity measurements were carried out using commercial systems Ecolume Biotest (Nera-S, Ltd., Moscow, Russia) and Biotox, (Nera-S, Ltd., Moscow, Russia).

### 2.10. Cultivation of Microbial Cells

Yeasts *Debaryomyces hansenii* VKM Y-2482 (*D. hansenii)* and *Blastobotrys adeninivorans* VKM Y-2677 *(B. adeninivorans)* provided by the All-Russian Collection of Microorganisms of IBFM RAS. For use with microorganized devices-the ES-20/60 injector (BioSan, Riga, Latvia), the TG16WS devices (Polycom, Inc., San Jose, CA, USA) and the Mini Spinplus (Eppendorf, Hamburg, Germany). The biomass was stored in micro–samples at a temperature of −25 °C. The following components were used to cultivate *D. hansenii* yeast and *B. adeninivorans*: glucose—1%, peptone—0.5%, yeast extract—0.05%. The cultivation time is 18–20 h (temperature 28 °C). Centrifugation was performed at 7000× *g* for 10 min. A phosphate-buffered solution (pH 6.8, 30 mm) was used to wash the biomass. For the formation of the receptor element 10 μL of suspension of microorganisms with a titer of 0.33 mg/L and 20 μL of PVA were mixed in a 1:2 ratio, and 10 μL of the mixture was applied to the electrode.

### 2.11. Using a 1st Generation Biosensor for Measurements

The BOD-thermoximeter EXPERT-009 (Econix-Expert LLC, Moscow, Russia) with a Clark electrode was used as a transducer for measuring the molecular oxygen content. A bioreceptor element with immobilized microorganisms was placed between the semi-permeable membrane of the electrode and the protective cap. To obtain a receptor element, microbial cells were added to 100 µL of PVA gel and shaken for 5 min. The resulting suspension was applied to a tablet with flat wells with a diameter of 5 mm and left to dry completely. During the measurements, a Clark electrode with a bioreceptor element was immersed in a 5 mL cuvette filled with 4 mL of phosphate-buffered solution with pH = 6.8. The cuvette was on a magnetic stirrer, and all experiments were carried out with constant stirring (200 rpm). After establishing a stable current level, a solution of the analyte was injected into the cell and the oxidation rate was recorded as the rate of change in current strength. Between each measurement, the cuvette and the electrode were rinsed with a buffer solution. The measurement temperature was 20 °C. The solutions were added using automatic micropipettes of variable volume (200–1000 µL, 20–200 µL) (Biohit, Helsinki, Finland).

### 2.12. Using a 2nd Generation Biosensor for Measurements

The working electrode of the mediator biosensor was formed by filling a plastic tube with a prepared paste “graphite powder–ferrocene–mineral oil”. The paste was prepared by mixing 10 mg of FC, 100 mg of graphite powder and 40 µL of oil. To modify the electrode, microbial cells were added to 100 µL of PVA gel and shaken for 5 min. The resulting suspension was applied to the electrode surface in a volume of 10 µL and dried at room temperature for 30 min. The layer of the immobilized biocatalyst was then covered with a dialysis membrane. To register the biosensor system signal, an electrochemical galvanopotentiostat converter was used, which registers the dependence of current strength on time—IPC Micro (NPO Volta, Moscow, Russia), to which the electrodes were connected. The measurements were carried out at a constant potential of 0.25 V. The measurements were carried out in a two-electrode system immersed in a measuring cuvette with a volume of 5 mL containing a potassium-sodium phosphate buffer (pH = 6.8). The reference electrode was saturated silver chloride. The cuvette was placed on a magnetic stirrer, and all experiments were carried out with constant stirring (200 rpm). After establishing a stable current level, a solution of the analyte was injected into the cell and the oxidation rate was recorded as the amplitude of the current strength. Between each measurement, the cuvette and the electrode were rinsed with a buffer solution. The measurement temperature was 20 °C. The solutions were added using automatic micropipettes of variable volume (200–1000 µL, 20–200 µL) (Biohit, Helsinki, Finland).

### 2.13. Determination of BOD by Standard Dilution Method

The dilution method was used as the reference method for determining BOD_5_. The analysis was conducted in accordance with the procedures outlined in methodology [28]. The dissolved oxygen content was determined using a BOD thermoximeter EXPERT-001-4.0.1 (Econix-Expert LLC, Moscow, Russia).

## 3. Results

### 3.1. Synthesis and Investigation of the Chemical and Spatial Structure of the PVA Polymer

**Figure 2:**
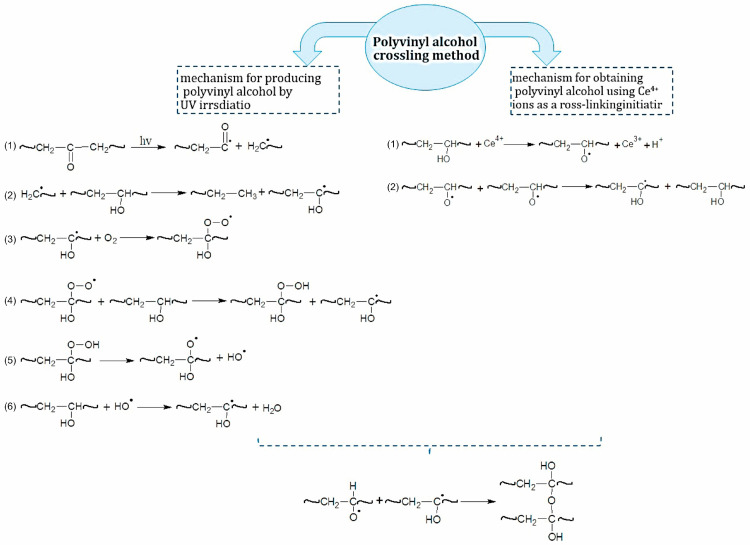
The mechanism of PVA hydrogel obtained by two modification methods.

In this work, we used two modification methods to obtain a mesh polymer: 1. PVA undergoes structural changes under the influence of ultraviolet radiation. The primary processes in polymers that occur during irradiation depend on the nature of the polymer and the chromophore group. PVA belongs to a group of polymers that degrade (i.e., macromolecules are destroyed) under the action of radiation only in dilute solutions (<0.3%). When radiation is applied to more concentrated solutions, cross-linking reactions occur. PVA, obtained from polyvinyl acetate, contains a certain amount of acetate and carbonyl groups [29]. Carbonyl groups belong to the chromophoric group (λ = 279, 285 nm), and when irradiated with light of wavelength 254–330 nm they readily enter an excited state. Subsequently, the process of breaking chemical bonds proceeds according to the Norrish mechanism [30]. The reaction proceeds through a radical mechanism and leads to chain breakage. 2 We used (NH_4_)_2_[Ce(NO_3_)_6_] as an initiator for radical polymer crosslinking. Figure 2 demonstrates the mechanism of formation of a mesh polymer obtained by using these methods.

The quality of the crosslinked polymers was assessed by their relative viscosity, which is an indication of the degree of polymerization. The relative viscosity of PVA-Ce^4+^ was 10.2 ± 0.2 and that of PVA-UV was 8.7 ± 0.2. The proportion of crosslinked polymer in the samples was determined through extraction. For PVA-Ce^4+^, this was 49.0 and for PVA-UV it was 42.0. The ability of the polymers to swell was assessed through the degree of swelling, which is the amount of liquid absorbed by the polymer compared to its mass. PVA-Ce^4+^ degree of swelling was 1.4 ± 0.1 and that of PVA-UV was 1.5 ± 0.1. Based on the kinetic swelling curves, it can be concluded that initial PVA belongs to polymers with unlimited swelling and PVA crosslinked with oxidative crosslinking belongs to polymers that have rapid, limited swelling with a low margin of swelling. This fact can be explained by the mesh structure of the crosslinked PVA, which means that even with an increase in temperature, the swelling will remain limited [31,32]. Since the hydrogel has a limited swelling capacity, it can be used in long-term analysis of real samples in buffer systems (Section S1; Figure S1).

**Figure 3:**
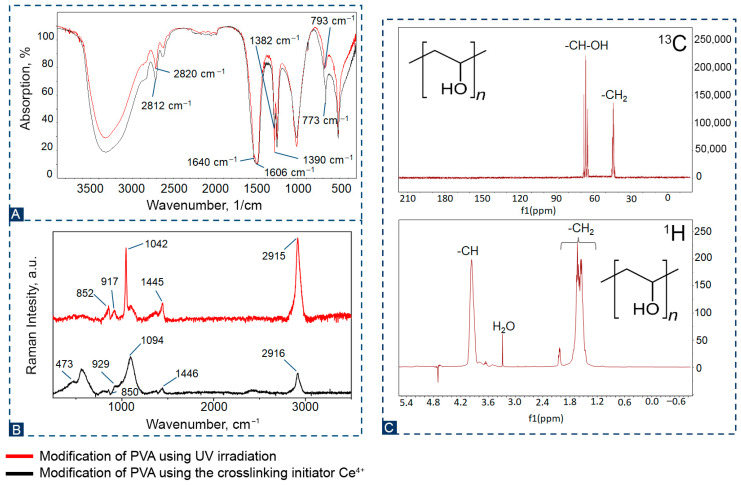
(**A**)—IR spectra of the obtained PVA; (**B**)—Raman spectra of the obtained PVA; (**C**)—^1^H and ^13^C NMR of the PVA hydrogel.

The chemical structure of PVA was studied using IR, Raman, and NMR spectroscopy (Figure 3). According to the IR spectrum shown in Figure 3A, it can be seen that the structure of PVA did not change when using two different modification methods. All the main peaks associated with hydroxyl and acetate groups are present on the spectra, as well as large bands associated with O–H stretching from intermolecular and intramolecular hydrogen bonds between 3670 and 3580 cm^−1^. The bands observed at 2820, 2812, 1640, and 1606 cm^−1^ refer to C-H stretching, C–O stretching from acetate groups, and C–O vibrations of aliphatic groups. The absorption bands of medium intensity at wave values of 1382 and 1390 cm^−1^ correspond to the deformation vibrations of the -CH group. Figure 3B shows Raman spectra of PVA with various modifications. Most pronounced Raman spectra and their corresponding designations have been confirmed by literature data [33,34,35]. The peaks at 2916 and 2951 cm^−1^ on the spectral line correspond to stretching fluctuations of the -CH and -CH_2_ groups. A shift of 1446 and 1451 cm^−1^ reveals low-intensity peaks associated with the bends in the -CH_2_ and -OH groups. According to the literature, a peak at 1094 and 1042 cm^−1^ is observed in the spectral line that is responsible for valence asymmetric oscillations of -C-OH. Fluctuations at 929, 850, 917 and 852 cm^−1^ correspond to stretching of -C-C groups, and fluctuations of 473 correspond to bending of -C=O and -CH groups outside the plane. Figure 3C shows the spectral characteristics in the ^1^H PVA NMR spectrum. Signals corresponding to methylene (-CH_2_-CH_2_-) and methyl (-CH-O-) protons in the main polymer chain are observed. For example, in D_2_O, these signals are manifested in the range of 1.62 ppm for methylene and 3.96 ppm for methyl protons. The NMR spectra shown in Figure 3C have a separate signal attributed to carbon in -CH-OH groups, which is located at 65 ppm. The quartet peak at 45 ppm is attributed to -CH_2_. These spectra are consistent with literature data [36].

**Figure 4:**
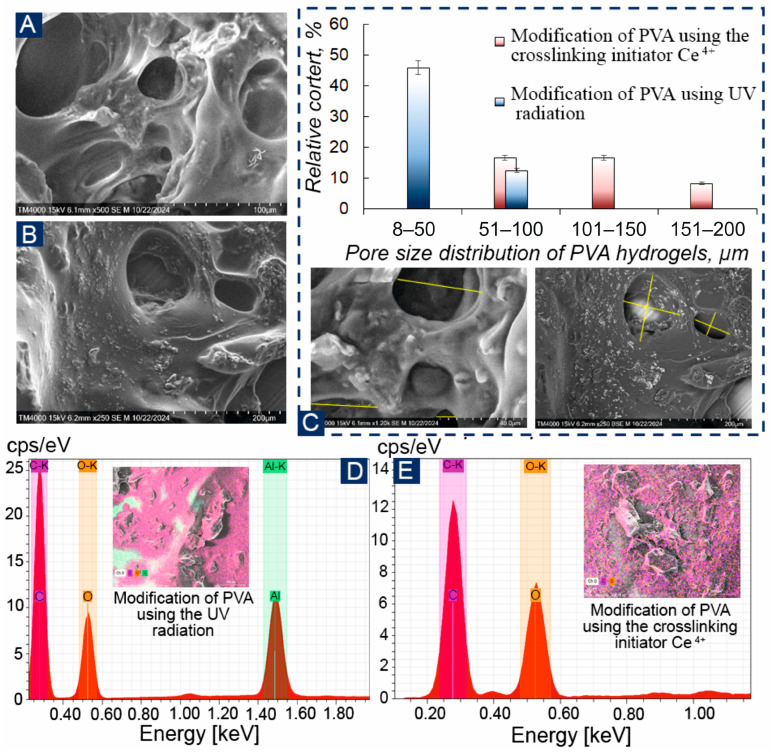
SEM hydrogel PVA: (**A**)—modification of PVA by UV irradiation; (**B**)—Modification of PVA using a crosslinking initiator Ce^4+^; (**C**)—pore size distribution of PVA hydrogels obtained by two methods; (**D**,**E**)—EDX for the obtained hydrogels.

The 3D architecture of the obtained PVA polymers was studied using scanning electron microscopy (Figure 4A,B and Figure S2, Section S2). In the SEM images, it can be seen that PVA has a porous structure, which allows it to retain microorganisms on the surface of electrodes and prevent them from leaching. When analyzing energy dispersive X-ray spectroscopy, the no detectable levels of Ce^4+^ above the instrument’s detection limit were found, and the distribution spectrum of the elements corresponds to modified UV, and aluminum was detected due to the fixation of the samples on the aluminum table (Figure 4D,E). Figure 4C shows a histogram of pore size distribution, and it can be observed that UV-irradiated PVA had the largest number of pores up to 50 μm in diameter, while Ce^4+^-crosslinked PVA had more pores between 50–100 μm. UV crosslinking creates pores through photoinitiated radical reactions with spatial variations in radical generation and polymer mobility, causing heterogeneous pore distributions. Ce^4+^ crosslinking generally yields more homogeneous oxidative radical formation throughout the matrix, resulting in denser and more uniform pore structures.

Pore size distribution in PVA hydrogels significantly impacts biosensing performance by controlling analyte diffusion, immobilization of microorganisms, mass transport, response time, sensitivity, and signal stability [37,38]. One of the most important factors for PVA polymer is its physicochemical characteristics, so, for the majority of gels described in the literature, good mechanical strength can be achieved only through complex chemical modification [39,40] (Sections S3 and S4; Figures S4 and S5, Table S1). In this case, for PVA hydrogels obtained by two modification methods, the deformation and strength characteristics were determined. Specifically, the maximum load, ultimate strength, elongation, bearing capacity of the material, and Young’s modulus were measured. The calculation of these deformation and strength parameters is presented in Table 1.

In general, as can be seen from the table, the physicomechanical characteristics of the hydrogels obtained by the two modification methods are similar. This was expected, since the stitching mechanisms are similar and differ only in the method of modification. However, with regard to ultimate strength, the PVA-Ce^4+^ hydrogel is slightly superior to the PVA-UV hydrogel, making it more promising for use as a receptor element in sensor arrays. The results obtained are consistent with data on the degree of cross-linking and swelling of synthesized polymers, indicating the superior characteristics of PVA Ce^4+^ hydrogels.

### 3.2. Physiological, Biochemical, Metabolic, and Biocatalytic Characteristics of Microorganisms Synthesized in Gels

**Figure 5:**
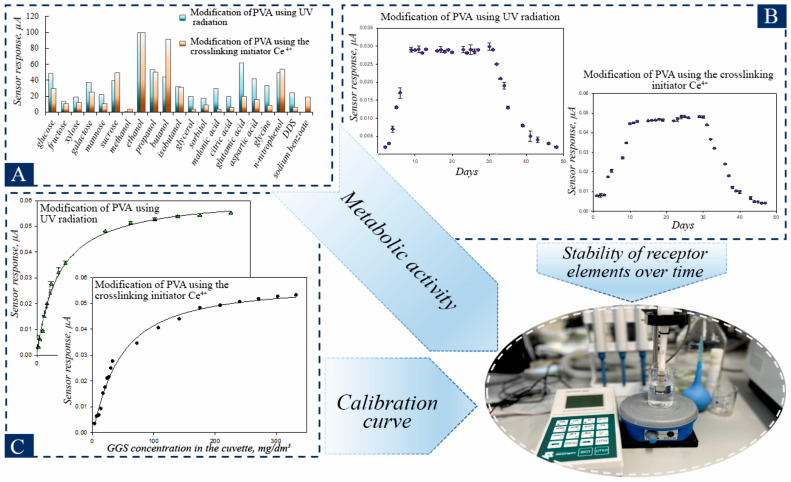
Characteristics of receptor elements based on PVA hydrogels and yeast *D. hansenii*: (**A**)—Metabolic activity of *D.hansenii* for hydrogels obtained by two modification methods; (**B**)—Stability of receptor elements over time; (**C**)—Calibration curve of activity from the concentration of glucose-glutamate mixture (GGM) in the cell.

Hydrogels were obtained by two methods: modification of PVA by UV treatment and modification of PVA using the initiator Ce^4+^ cross-linking to form receptor elements. The potential toxicity of the synthesized polymers was evaluated using a bacterial bioluminescence inhibition assay. Contrary to exhibiting inhibitory effects, the tested materials were found to stimulate bacterial luminescence. This result indicates the non-toxic and biocompatible nature of the polymer matrix, a prerequisite for its safe application as an immobilization support for living cells. The yeast *D. hansenii* VKM Y-2482, was used as a recognizing element, which has wide substrate specificity and high resistance to heavy metals and was previously used effectively to create BOD biosensors [41,42]. The proposed approach to selecting a receptor element is based on a comparative analysis of the sensitivity of microorganisms to oxidizable organic substrates. The metabolic activity of yeast *D. hansenii* was evaluated for hydrogels produced by two modification methods (Figure 5A).

For the purpose of BOD_5_ analysis, it is crucial that the developed biosensor can detect the biochemical oxidation of a diverse array of natural substrates. The BOD index is a comprehensive indicator that encompasses organic substances present in water. To guarantee the utmost precision in BOD analysis, it is essential to provide microorganisms with a wide variety of oxidizable substrates. Consequently, the substrate specificity of microorganisms within the constructed frameworks was investigated. It can be seen from the data in Figure 5A that, for *D. hansenii* with different modifications of PVA, the maximum response was observed to ethanol, which was assumed to be 100%. It should be noted that yeast are good at oxidizing monosaccharides and disaccharides (galactose, sucrose, xylose, and mannose), as well as some lower alcohols being partially oxidized by these bacteria. It should also be noted that, when modifying PVA using Ce^4+^ initiator for crosslinking, there is a decrease in response to carbohydrates during the transition from aldose (glucose) to ketose (fructose) and the oxidation of disaccharide (sucrose) is less intense than that of monosaccharide. Thus, immobilization of *D. hansenii* yeast into PVA hydrogels obtained by two methods of modification has almost no effect on metabolic activity, and results obtained correlate well with those published for other methods for immobilizing these yeasts [42].

Physico-biological and biocatalytic characteristics were determined for yeast-based receptor elements (Figure 5B,C, Figures S6 and S7 (Section S5), Table 2). A mixture of glucose and glutamic acid in a 1:1 mass ratio GGM was used as a model solution, which is used as a standard in the definition of BOD_5_ in international practice [28].

The developed receptor elements based on PVA and *D. hansenii* hydrogels have similar physico-biological and biocatalytic properties, which again confirms that PVA is an excellent biocompatible polymer for the inclusion of microorganisms. However, comparative analysis has shown that PVA-Ce^4+^ is superior to other polymers in terms of mechanical strength, which is important for future practical application of the receptor elements. Additionally, the approach proposed in this work for obtaining PVA hydrogels by radical modification using Ce^4+^ is very simple and inexpensive, does not require additional reagents or synthesis steps to achieve a high degree of cross-linking, which also makes it very important from a practical point of view. Therefore, further experiments were conducted using the PVA-Ce^4+^ hydrogel.

### 3.3. The Use of PVA Hydrogel in Biosensors for Rapid Analysis of Water Pollution

The BOD index is a quantitative assessment of the ability of wastewater to deplete oxygen reserves. The standard method of determination is based on incubating the sample for at least 5 days. However, the danger of a 5-day analysis lies in its inability to accurately assess the real threat of bio-oxidizable organic impurities entering a natural reservoir in case of an accident at a sewage treatment plant. Consequently, it is impossible to take timely measures to eliminate the consequences until standard analysis is complete. Kinetic approaches to BOD assessments, implemented in microbial biosensors, reduce analysis time to a few minutes [43].

To quantify the BOD index in the sample, mediator biosensors based on ferrocene (FC) and neutral red (NR) with immobilized microorganisms were formed. This system has already proven to be the best for transferring electrons using yeast accounts [44]. Yeasts *D. hansenii* and *B. adeninivorans* were used as biocatalysts. These microorganisms are characterized by resistance to high temperatures, high concentrations of metal ions, and high osmotic pressure, as well as a large number of metabolizable substrates, including *B. adeninivorans* and *D. hanssenii* [27,45]. Based on previous studies described in [27], FC outperforms other used mediators in terms of electron transfer rate to the graphite paste electrode (the rate constant for heterogeneous electron transfer is 0.4 ± 0.1 cm/s [46] and 400 mV redox-potential relative to the standard hydrogen electrode (pH = 7.0) [47]). Therefore, it was chosen to be one of the components of the system. A NR mediator was also used as a second mediator to form the receptor system because it has the highest interaction rate constant with the eukaryotic cells under study (0.681 ± 0.009 dm^3^/(g·s) with *B. adeninivorans* [27] and redox-potential −330 mV relative to the standard hydrogen electrode (pH = 7.0) [48].

**Figure 6:**
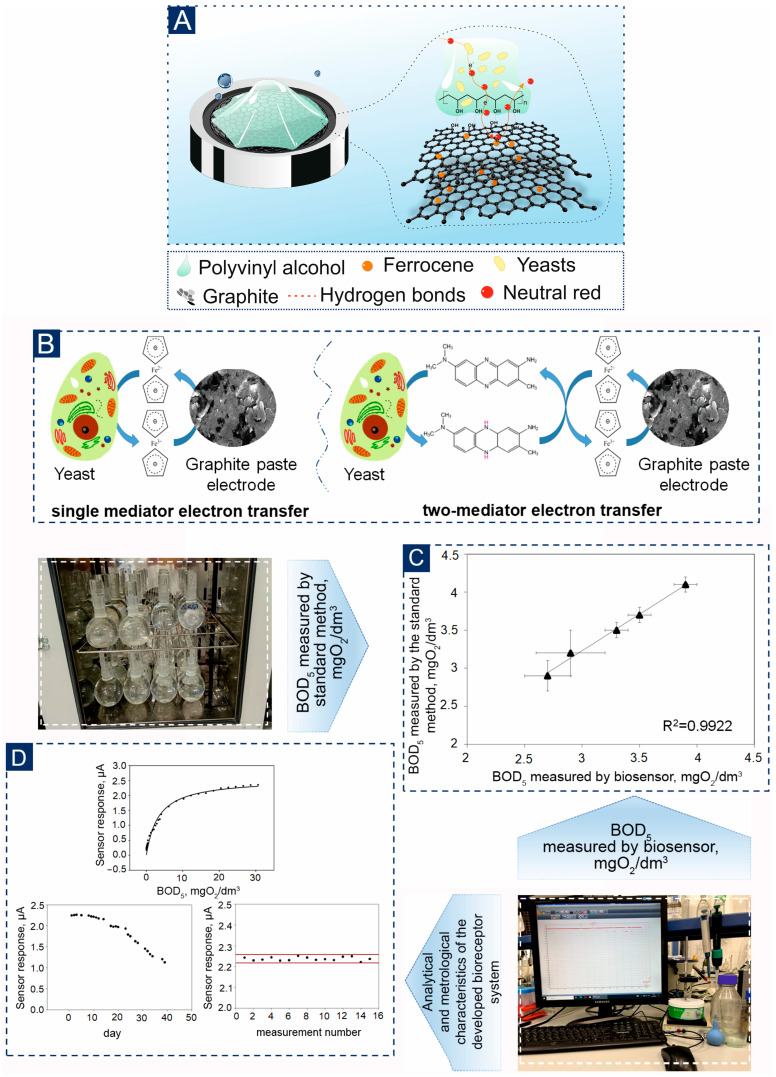
Characteristics of a bioreceptor element based on the PVA-Ce^4+^ hydrogel and *B. adeninivorans* yeast: (**A**)—Mechanism of orientation of the bioreceptor element on the surface of the graphite-paste electrode (**B**)—Mechanism of the one- and two-mediator system; (**C**)—Analysis of water samples by a developed biosensor and a standard method for determining BOD_5_; (**D**)—Analytical and metrological characteristics of the developed bioreceptor system.

One of the effective approaches to increase electron transfer is the use of two-mediator systems [49,50]. In such a system, one mediator must quickly interact with the microorganism, providing an exchange of electrons, while the second mediator performs the function of transferring electrons to the electrode (Figure 6A,B). This principle helps to increase the sensitivity and speed of analysis, which can be especially useful in biosensors and other analytical devices. It should be noted that our research group has previously demonstrated the effectiveness of *D. hansenii* yeast combined with mediators FC and methylene blue [41]. As a result, it was found that the lower limit for the determined BOD_5_ concentration for the biosensor was 2.5 mg/dm^3^. In contrast, in a single-mediator system, determination was possible only at a concentration of 5.1 mg/dm^3^ using a NR mediator. These improvements allow for more accurate and efficient measurement of biochemical parameters, opening up new possibilities for use in environmental monitoring and other fields. The calibration dependences of the analytical signal on the BOD index were obtained for these biosensors (Figure 6C,D, Figures S8 and S9). An important characteristic of a biosensor is its operational stability. This indicates how consistent the sensor’s response is to the same substrate concentration over a large number of consecutive measurements. The operational stability of a biosensor is characterized by the change in its activity (as a percentage of its initial value) after multiple consecutive measurements over a specific time period. The operational stability of the developed biosensors is illustrated in the following Figure 6D.

The modified *t*-test was employed for statistical evaluation of the data: there were no significant disparities between the data obtained through the two distinct methods (Figure 5C). The developed biosensors can serve as for BOD_5_ monitoring instruments [44]. The electrochemical response of the bioreceptor element based on whole cells is provided by enzymatic reactions of microorganisms. Modeling within the framework of Michaelis-Menten kinetics allows us to use Equation (2) to approximate obtained curves.

(2) $$R=\frac{R_{max}[S]}{K_{M}+[S]}$$

where *R_max_*—the maximum rate of the enzymatic reaction achieved by [*S*], *K_M_*—the effective Michaelis constant, i.e., the substrate concentration at which *R* = *R_max_*/2.

Equation (2) implies that, at low substrate concentrations, the analytical signal is proportional to BOD_5_. This makes it possible to identify a linear section of the calibration curve bounded from above by the K_M_ value. The lower boundary of this linear section is calculated statistically based on a criterion of relative standard deviation for measurement results (Sr(C) < 0.33). Table 3 presents the main analytical and metrological characteristics of BOD biosensors.

Based on the analysis of metrological and analytical characteristics presented in Table 3, we can conclude that the biosensor’s response is more stable when using the «FC+NR—*B. adeninivorans*—PVA-Ce^4+^» system, with a relative standard deviation of 0.45%. As for long-term stability, all biosensors last about 30 days on average, which is sufficient for laboratory testing (Figure 6D and Figure S9). The time taken for one analysis does not exceed 16 min, significantly increasing the analysis efficiency compared to the traditional BOD_5_ method. For the analysis of natural waters, it is most advisable to use the «FC+NR–*B. adeninivorans*—PVA-Ce^4+^» system, since the lower limit of the biosensor allows estimating BOD_5_ values in the range from 0.10 to 3.81 mgO_2_/dm^3^.

Table 4 also shows the comparative characteristics of known analogues, it should be noted that the developed biosensor has a wider linear range (0.1–3.81 mgO_2_/dm^3^) compared to analogues, which makes it possible to determine both low and moderate concentrations of BOD with high accuracy. Unlike *Bacillus subtilis*, the developed system based on yeast *B. adeninivorans* exhibits higher sensitivity and response time. This can be attributed to the permeability of the covalently cross-linked PVA matrix where Ce^4+^ acts as a cross-linking initiator.

The increase in sensitivity and stability is significantly influenced by the uniform orientation of the receptor element along the surface of the graphite paste electrode (Figure 5A). This orientation is due to the formation of hydrogen bonds between hydroxyl groups in the composition of PVA and OH groups on the surface and in graphite defects [55,56]. These factors together accelerate the kinetics of processes in the near-electrode space, contributing to the diffusion of the mediator.

Thus, the «FC+NR—*B. adenivorans*-PVA-Ce^4+^» system surpasses known analogues in key parameters such as sensitivity, selectivity and stability, making it the optimal choice for creating highly efficient biosensors for BOD.

5 wastewater samples were collected to test the newly developed biosensor. Figure 5C illustrates the correlation between BOD values obtained using the biosensor and those obtained by the standard dilution method for water samples. The results from the analysis using the standard dilution technique and the biosensor method differ slightly from one another. Therefore, the biosensor based on *B. adeninivorans* cells immobilized in PVA and the system of FC-NR mediators is an effective tool for analyzing various water samples.

## 4. Conclusions

The modification of PVA was carried out under the influence of UV irradiation and in the presence of the redox initiator cerium-ammonium nitrate. These methods have been shown to improve the physico-chemical properties of the polymer, increasing its stability and mechanical strength. The structure of the modified PVA was determined using infrared, Raman, and NMR spectroscopy. These techniques revealed that crosslinking forms a three-dimensional network, which helps the material retain water and improves other functional characteristics. This modified PVA has potential applications in various biotechnologies, such as the creation of biosensors and analytical devices.

The analysis of the analytical and metrological characteristics of bioreceptor elements based on *D. hansenii* yeast and PVA hydrogels has been conducted. It has been demonstrated that modified PVA, with the help of an initiator for Ce^4+^ cross-linking, makes it possible to produce bioreceptor elements with excellent characteristics. Biosensors with mediators based on FC and NR were formed using immobilized yeast cells of *B. adeninivorans* and *D. hansenii*, and a film of modified PVA-Ce^4+^ was used. It has been revealed that «FC+NR-*B. adeninivorans*—PVA-Ce^4+^» is the best system, as the lower limit of the biosensor it creates allows estimating BOD_5_ values as low as 0.10 mgO_2_/dm^3^, and the sensor itself has very high operational stability. The analysis of surface water samples was performed using a biosensor based on *B. adeninivorans* yeast, a two-mediator system, and a modified PVA. The results obtained differ slightly from the results obtained by the standard BOD determination method, which makes it possible to use the created biosensor as a prototype of a serial device for an alternative method of rapid determination of water quality.

## Figures and Tables

**Table 1:** Physico-mechanical properties of hydrogels obtained by two modification methods.

Samples	Width b, mm	Cross-Sectional Area S0, mm^2^	Destructive Stress, Fp, H	Ultimate Strength, MPa	Relative elongation, ε, %	Bearing Capacity of the Material, F_H_c, H/m	Young’s Module, E, MPa
PVA-Ce^4+^	20	1.0	57 ± 5	70 ± 4	45 ± 6	285 ± 4	3500 ± 150
PVA-UV	20	1.0	52 ± 4	55 ± 6	50 ± 3	270 ± 3	3400 ± 150

**Table 2:** Comparative characteristics of the obtained receptor elements based on hydrogels of PVA and yeast *D. hansenii*.

Parameters of the Receptor Element	PVA-UV	PVA-Ce^4+^	PVA+N-VP [42]
Stability, day	48	45	42
Relative standard deviation of measurement, %	2.4	2.0	2.0
Sensitivity coefficient, s^−1^	0.007 ± 0.001	0.008 ± 0.004	0.005 ± 0.001

**Table 3:** Characteristics of developed biosensors.

Characteristic	Developed Biosensor					
	*B. adeninivorans*			*D. hansenii*		
	FC	NR	FC+NR	FC	NR	FC+NR
Operational stability, %	5.08	1.64	0.45	5.37	3.87	2.04
Long-term stability, day	32	30	37	31	29	35
Duration of a single measurement, min.	15–16	15–16	15–16	15–16	15–16	15–16
Linear range of detectable BOD concentrations, mgO_2_/dm^3^	3.03–10.35	1.98–17.71	0.10–3.81	4.47–18.35	2.77–21.25	1.84–5.07

**Table 4:** Comparison with literary analogues.

Microorganisms	System	Linear Range (mgO_2_/dm^3^)	Sr, %	Analysis Time, min.	References
*B. adeninivorans*	FC-NR–PVA-Ce^4+^	0.1–3.81	0.45	<5	This work
*D. hansenii*	FC-Methylene Blue	2.5–7.2	1.2	<10	[49]
Active sludge	-	25–500	4.3	<10	[51]
*S. cerevisiae*	potassium ferricyanide + vitamin K3	20–225	4.16	20	[52]
*B. adeninivorans*	FC-NR	0.16–2.7	1.5	<5	[27]
*Bacillus subtilis*	PVA + sodium alginate	10.5–210	<16.42	8	[53]
*P. yeii*	Xerogel-PHB	0.5–50	5.4	5	[54]

## Data Availability

The data that support the findings of this study are available on request from the corresponding author.

## Supplementary Materials

The following supporting information can be downloaded at: https://www.mdpi.com/article/10.3390/bios16010042/s1. The attached file contains detailed data on PVA-based hydrogels divided into several numbered sections, tables, and figures. Section S1: Kinetics of swelling of PVA-based hydrogels in water; Figure S1: Kinetics of swelling of PVA-based hydrogels in water; Section S2: Scanning electron microscopy of PVA hydrogel; Figure S2: Scanning electron microscopy of PVA hydrogel: (A–C) modification of PVA using UV irradiation; (D–E) modification of PVA using the cross-linking initiator Ce^4+^; Section S3: Physical and mechanical properties of hydrogels obtained by two modification method; Table S1: Characteristics to be determined; Figure S3: Force vs. Displacement Graph: (A) Modification of PVA using UV irradiation; (B) Modification of PVA using the crosslinking initiator Ce^4+^; Figure S4: Stress vs. displacement graph: (A) Modification of PVA using UV irradiation; (B) Modification of PVA using the crosslinking initiator Ce^4+^; Section S4: Physiological, biochemical, metabolic and biocatalytic characteristics of microorganisms in synthesized gel; Figure S5: Linear section of the calibration dependence of the biosensor responses on the concentration of GGS in the cuvette: (A) Modification of PVA using UV irradiation; (B) Modification of PVA using the crosslinking initiator Ce^4+^; Figure S6: Operational stability: (A) Modification of PVA using UV irradiation; (B) Modification of PVA using the crosslinking initiator Ce^4+^; Section S5: Use of PVA hydrogel in biosensors; Figure S7: Calibration dependencies for the systems: (A) FC—*B.adeninivorans*-PVA-Ce^4+^; (B) NR—*B. adeninivorans*-Ce^4+^; (C) FC+NR—*B. adeninivorans*-PVA-Ce^4+^; (D) FC—*D. hansenii*-PVA-Ce4+; (E) NR—*D. hansenii*-Ce^4+^; (F) FC+NR—*D. hansenii*-PVA-Ce^4+^; Figure S8: Operational stability of biosensors: (A) FC—*B.adeninivorans*-PVA-Ce^4+^; (B) NR—*B. adeninivorans*-Ce^4+^; (C) FC+NR—*B. adeninivorans*-PVA-Ce^4+^; (D) FC—*D.hansenii*-PVA-Ce^4+^; (E) NR—*D. hansenii*-Ce^4+^; (F) FC+NR—*D. hansenii*-PVA-Ce^4+^; Figure S9: Long-term stability of biosensors: (A) FC—*B. adeninivorans*-PVA-Ce^4+^; (B) NR—*B.adeninivorans*-Ce^4+^; (C) FC+NR—*B.adeninivorans*-PVA-Ce^4+^; (D) FC—*D.hansenii*-PVA-Ce^4+^; (E) NR—*D.hansenii*-Ce^4+^; (F) FC+NR—*D.hansenii*-PVA-Ce^4+^.
