# Supplementary material for: Engineered PVA Hydrogel as a Universal Platform for Developing Stable and Sensitive Microbial BOD-Biosensors
Source: Biosensors (Basel). 2026 Jan 4;16(1):42. doi: 10.3390/bios16010042 (PMC12839383; doi:10.3390/bios16010042)
Supplement: Supplementary file 1 [file biosensors-16-00042-s001.zip › biosensors-4001992-supplementary.pdf]

Supporting Information for the article:

Engineered PVA Hydrogel as a Universal Platform for Developing Stable and Sensitive Microbial BOD-Biosensors

Anastasia Medvedeva[1,2], Aleksandra Titova[1], Anna Kharkova[1,2], Roman Perchikov[1], George Gurkin[1], Lyudmila Asulyan[2], Leonid Perelomov[3], Maria Gertsen[3], Vyacheslav Arlyapov[1,2*]

[1]Research Center "BioChemTech", Tula State University, Tula, Lenin Ave., 92, Tula 300012, Russia.
[2]Department of Chemistry, Tula State University, Tula, Lenin Ave., 92, Tula 300012, Russia.
[3]Laboratory of Biogeochemistry, Faculty of Natural Sciences, Tula State Lev Tolstoy Pedagogical University, Lenin Avenue, 125, Tula 300026, Russia

*Correspondence: v.a.arlyapov@tsu.tula.ru

Table of contents

Section S1. Kinetics of swelling of PVA-based hydrogels in water 2

Section S2. Scanning electron microscopy of PVA hydrogel 2

Section S3. Physical and mechanical properties of hydrogels obtained by two modification methods. 2

Section S4. Physiological, biochemical, metabolic and biocatalytic characteristics of microorganisms in synthesized gels. 4

Section S5. Use of PVA hydrogel in biosensors 5

Section S1: Kinetics of swelling of PVA-based hydrogels in water

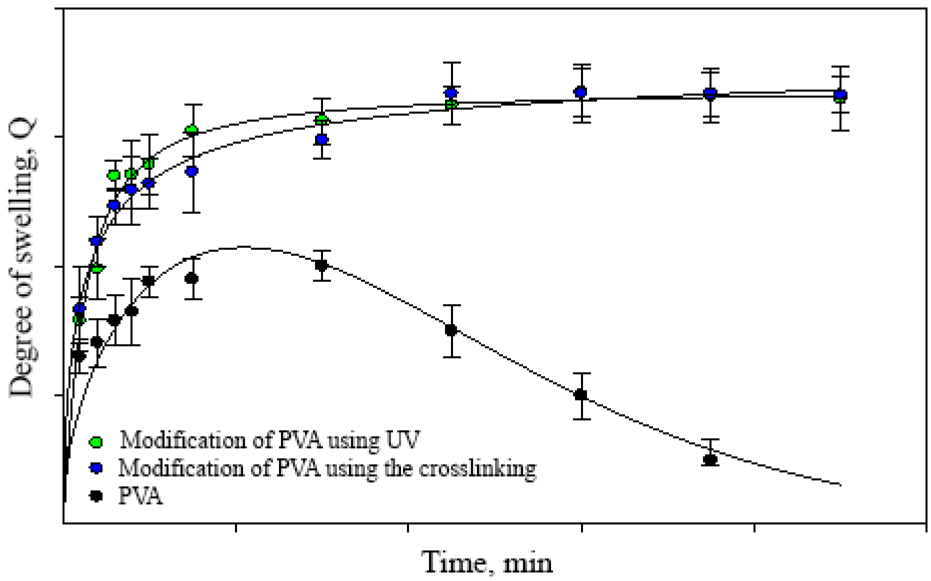

Figure S1: Kinetics of swelling of PVA-based hydrogels in water

Section S2: Scanning electron microscopy of PVA hydrogel

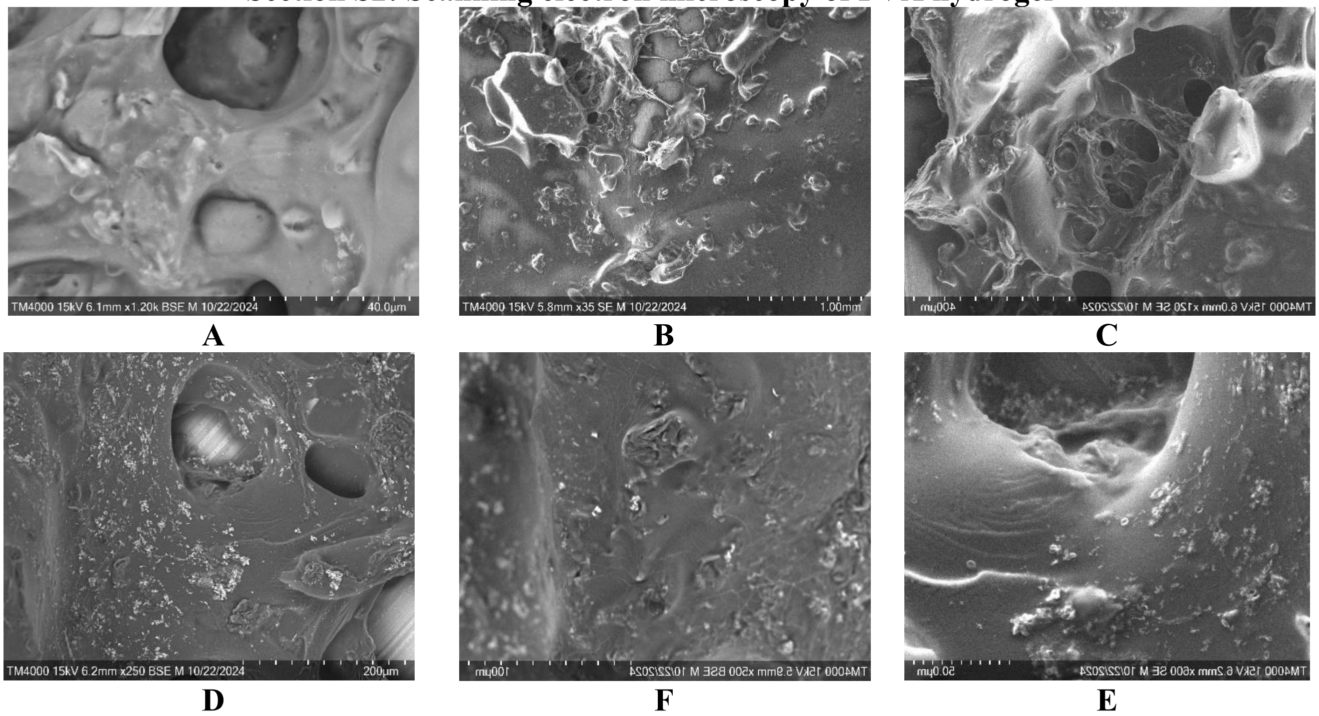

Figure S2: Scanning electron microscopy of PVA hydrogel: A-C) modification of PVA using UV irradiation; D-E) modification of PVA using the cross-linking initiator Ce^{4+}.

Section S3: Physical and mechanical properties of hydrogels obtained by two modification methods

To conduct tests aimed at determining the physical and mechanical characteristics, films measuring 20×2 cm were prepared in advance and dried at 250 for 24 hours. The resulting films were analyzed at the Tula State University Innovation Center on an Instron device in accordance with GOST 14236-81 (Polymer films. Tensile testing method).

Table S1: Characteristics to be determined.

Characteristic	Definition	Formula
Destructive stress F_p, N	The amount of force required to tear a material	-
Tensile strength σ_p, MPa	Mechanical stress above which destruction of the material occurs.	$\sigma_p = \frac{F_p}{S_0}$
Elongation at break, ε_p %	The ratio of the increase in the calculated length of a specimen after rupture to its initial value	$\varepsilon_p = \frac{\Delta l_p}{l_0} \cdot 100\%$
Young's modulus, MPa (modulus of elasticity)	Young's modulus shows the force with which a rod of unit cross-section must be compressed (or stretched) to obtain a change in length of 1 unit.	$E = \frac{F/S}{\Delta l / l} = \frac{Fl}{S\Delta l}$
Load-bearing capacity of the material, F_{ns}, N/m	The maximum load that a material can bear without losing its functional qualities.	$F_{нс} = \frac{F_p}{в}$

Determining the dependence of the load on the displacement of the clamps allows us to obtain a number of physical and mechanical characteristics: ultimate strength $\sigma_в$ ($\sigma_p = \frac{F_p}{S_0}$, MPa), breaking stress F_p (graphically, N), bearing capacity of the material: ($F_{нс} = \frac{F_p}{в}$, N/m)

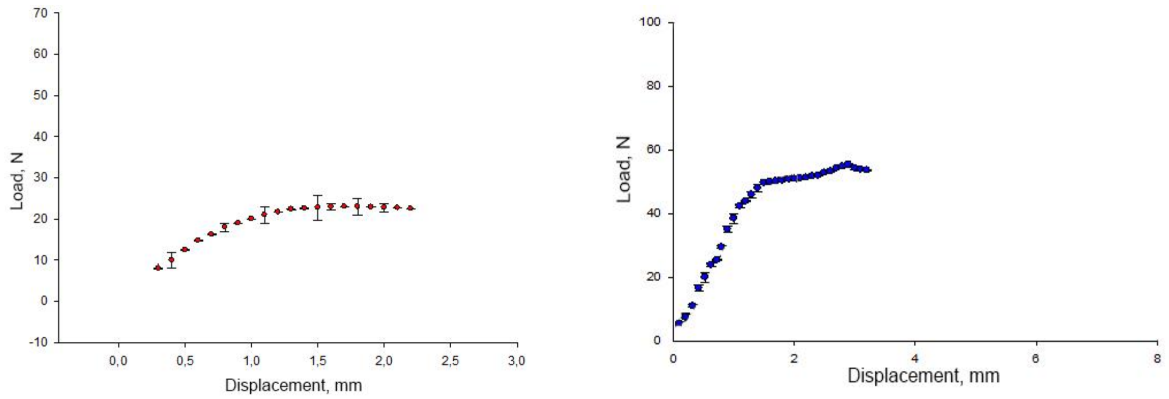

A B

Figure S3: Force vs. Displacement Graph: A) Modification of PVA using UV irradiation ;B) Modification of PVA using the crosslinking initiator Ce^{4+}

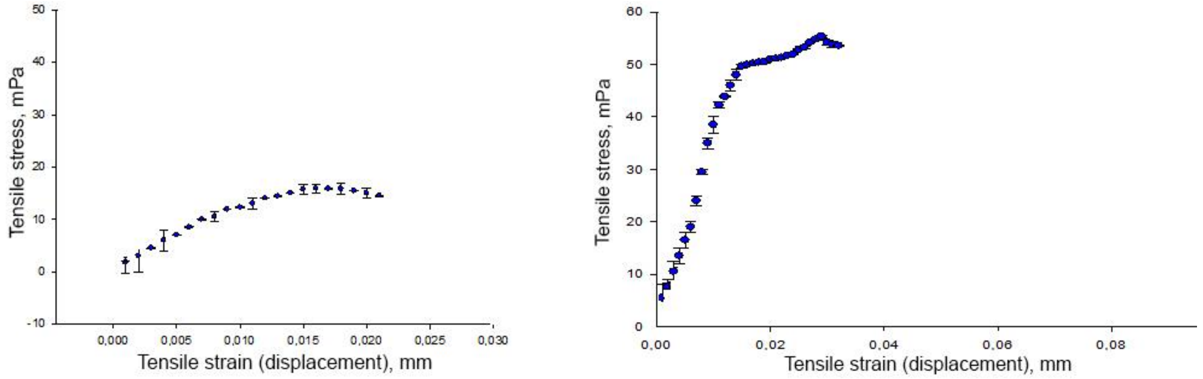

A **B**

Figure S4: Stress vs. displacement graph: A) Modification of PVA using UV irradiation; B) Modification of PVA using the crosslinking initiator Ce^{4+}

Section S4: Physiological, biochemical, metabolic and biocatalytic characteristics of microorganisms in synthesized gels

Physicobiological and biocatalytic characteristics were determined for receptor elements based on *D.hansenii* yeast. A mixture of glucose and glutamic acid in a mass ratio of 1:1 (GGA), which is used as a standard in determining BOD_5 in international practice, was used as a model solution.

Dependences of the intensity of the sensor response based on the obtained receptor elements on the concentrations of substrates in the cuvette

To reduce errors in the analysis of real samples, the linear section of the calibration curve is used. Working in the linear range significantly simplifies the calibration procedure of a real analyzer and, in addition, it is possible to carry out measurements with a minimum error. The linear range of the hyperbola is limited from below by the minimum detection limit, and from above by the substrate concentration equal to the Michaelis constant. The obtained data are presented in Figure S3.

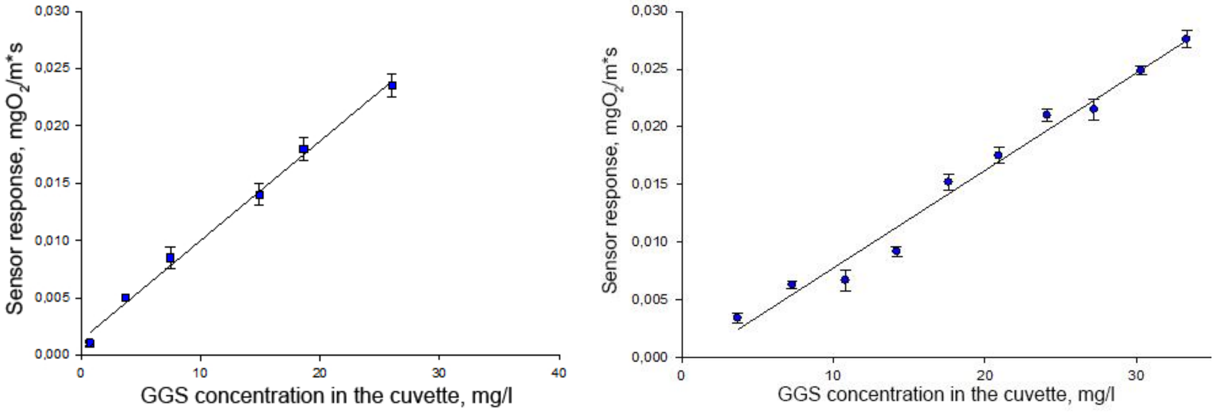

A **B**

Figure S5: Linear section of the calibration dependence of the biosensor responses on the concentration of GGS in the cuvette: A) Modification of PVA using UV irradiation; B) Modification of PVA using the crosslinking initiator Ce^{4+}

Operational stability of biosensors

Operational stability is closely related to the metrological characteristic of the method – convergence (repeatability). Figure S4 shows the operational stability of biosensors

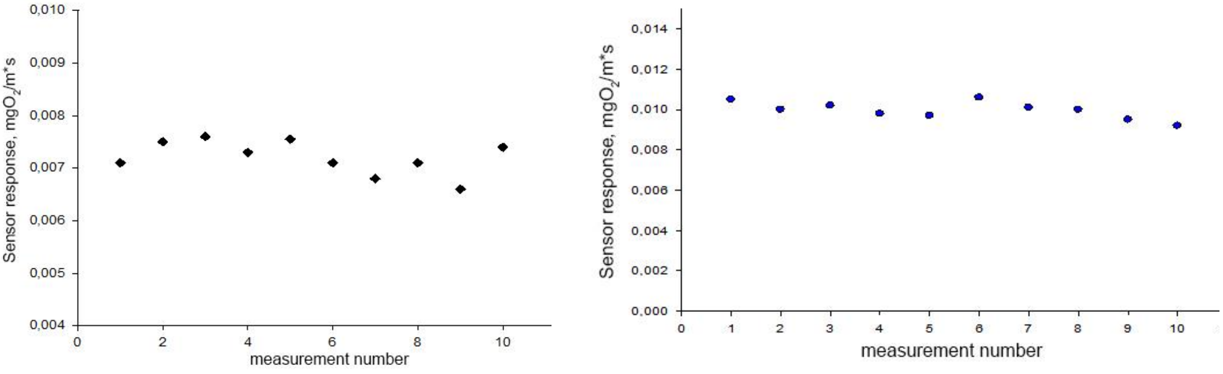

A **B**

Figure S6: Operational stability: A) Modification of PVA using UV irradiation; B) Modification of PVA using the crosslinking initiator Ce^{4+}

Section S5: Use of PVA hydrogel in biosensors

Testing of the developed bioelectrochemical system for BOD index determination

The calibration dependence of the biosensor response on the substrate concentration is the most important metrological characteristic, since in order to quantify the analytes in a sample, it is necessary to study the dependence of the analytical signal on the concentration.

Based on the data obtained during the experiment, calibration dependences of the analytical signal on the BOD_5 index were constructed for all systems (Figure S5).

Next, the dependence of the sensor response on BOD was approximated based on the Michaelis-Menten equation.

$$V = \frac{V_{\max}[S]}{K_M + [S]},$$

where V is the rate of the enzymatic reaction;

V_{max} is the maximum speed of the enzymatic reaction at which the entire enzyme participates in the formation of the enzyme-substrate complex;

K_M - apparent Michaelis constant - substrate concentration at which the reaction rate is half the maximum;

$[S]$ - BOD_5 value.

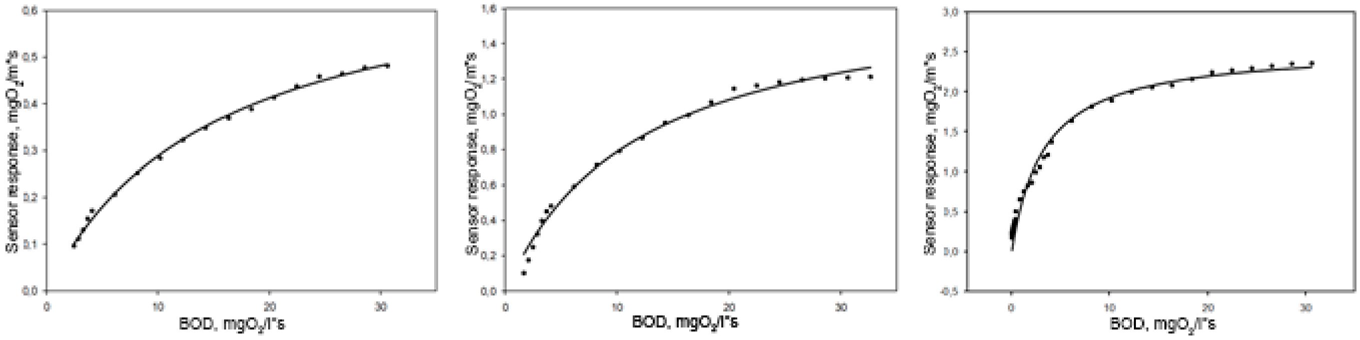

A **B** **C**

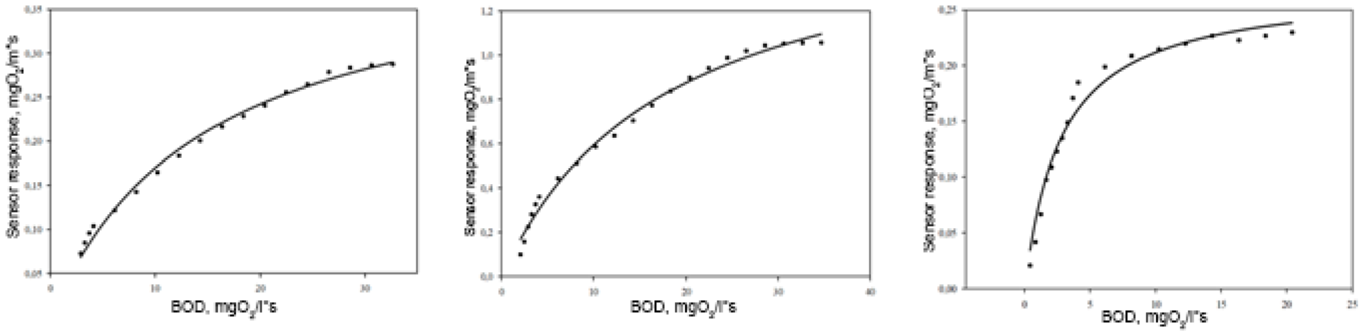

D E F

Figure S7: Calibration dependencies for the systems: A) FC – *B.adeninivorans* - PVA-Ce^{4+}; B) NR – *B.adeninivorans* - Ce^{4+}; C) FC+NR – *B.adeninivorans* - PVA-Ce^{4+}; D) FC – *D.hansenii* -PVA-Ce^{4+}; E) NR – *D.hansenii* - Ce^{4+}; F) FC+NR – *D.hansenii* - PVA-Ce^{4+}

Operational and long-term stability of biosensors

The stability of a biosensor is the most important characteristic of its operation. Operational stability is closely related to the metrological characteristic of the method - convergence (repeatability).

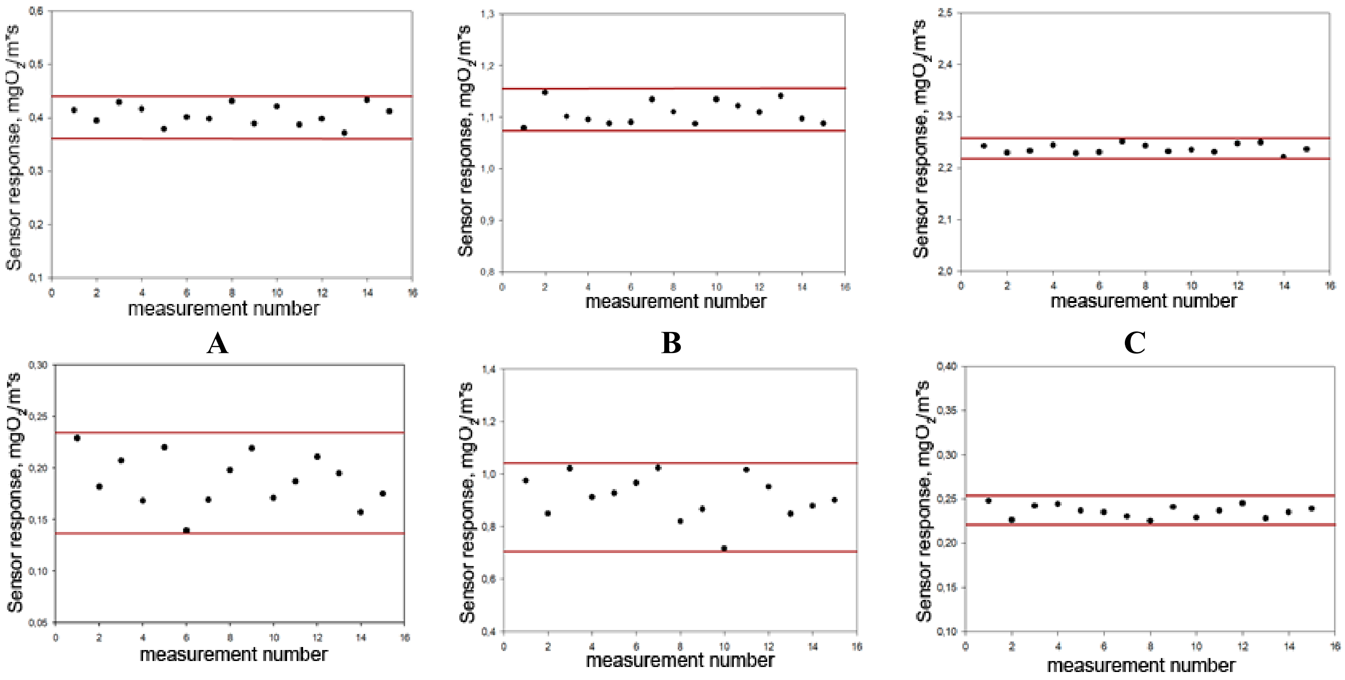

D E F

Figure S8: Operational stability of biosensors: A) FC – *B.adeninivorans* -PVA-Ce^{4+}; B) NR – *B.adeninivorans* - Ce^{4+}; C) FC+NR – *B.adeninivorans* - PVA-Ce^{4+}; D) FC – *D.hansenii* -PVA-Ce^{4+}; E) NR – *D.hansenii* - Ce^{4+}; F) FC+NR – *D.hansenii* - PVA-Ce^{4+}

Long-term stability characterizes the stability of the sensor over a long period of time. The time during which the signal value was at least 50% of the maximum activity is taken as the time of stable operation of the receptor element. Long-term stability was determined by daily measuring the magnitude of the sensor response to the same concentration of a solution of a glucose and glutamic acid mixture. The sensor was stored

in a buffer solution at a temperature of +4°C between measurements. The obtained dependencies are shown in Figure S7.

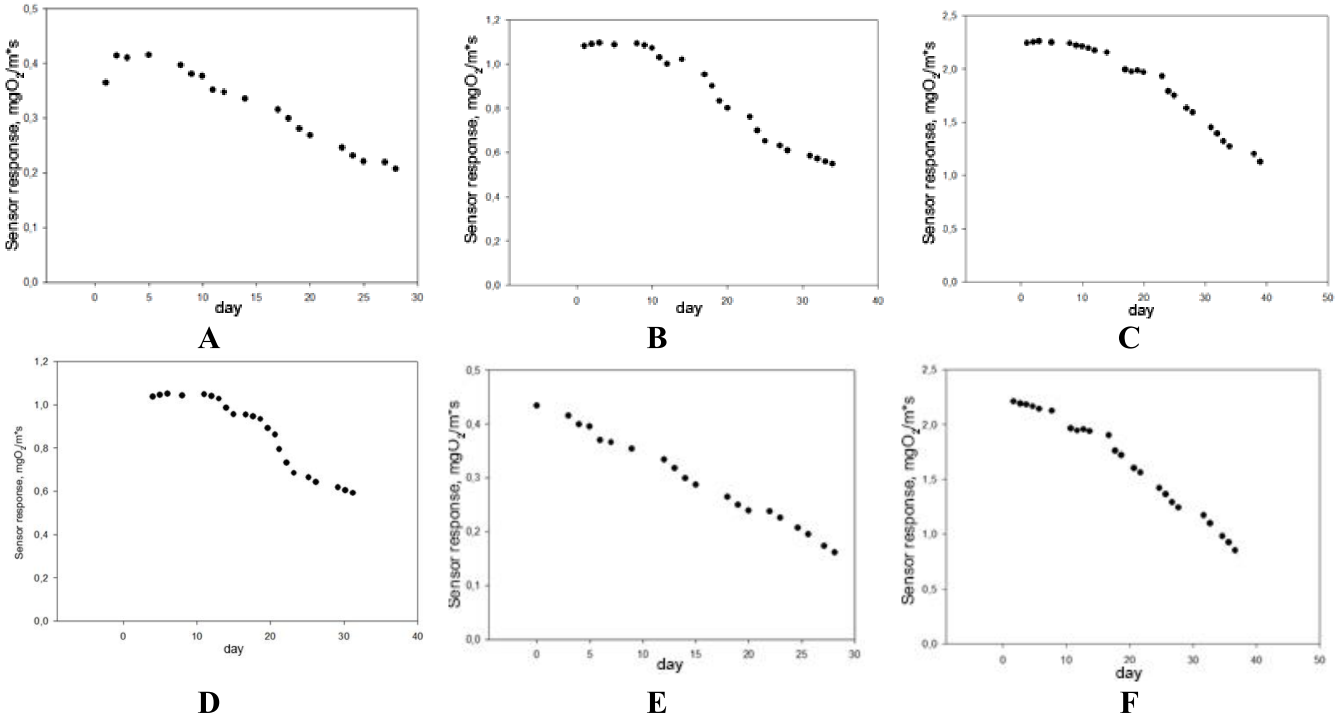

Figure S9: Long-term stability of biosensors: A) FC – *B.adeninivorans* -PVA-Ce^{4+}; B) NR – *B.adeninivorans* - Ce^{4+}; C) FC+NR – *B.adeninivorans* - PVA-Ce^{4+}; D) FC – *D.hansenii* -PVA-Ce^{4+}; E) NR – *D.hansenii* - Ce^{4+}; F) FC+NR – *D.hansenii* - PVA-Ce^{4+}
